# Aqueous extract of *Tamarindus indica *fruit pulp exhibits antihyperglycaemic activity

**Published:** 2020

**Authors:** Rajesh Nivesh Krishna, Roy Anitha, Devaraj Ezhilarasan

**Affiliations:** 1 *Department of Pharmacology, Saveetha Dental College and Hospitals, Saveetha Institute of Medical and Technical Sciences (SIMATS), Tamil Nadu, India*

**Keywords:** Tamarindusindica, Anti-diabetic, Cytotoxicity, Glucose uptake

## Abstract

**Objective:**

*Tamarindus indica *Linn. (*T.indica*) is a well-known plant used in traditional medicine. The plant is popular for its antidiabetic activity. However, effect so f its aqueous fruit pulp extract on carbohydrate hydrolyzing enzymes and its glucose uptake potential were not explored.

**Materials and Methods:**

The antidiabetic activity was assessed by *in-vitro *α-amylase and α-glucosidase inhibitory assays after preliminary phytochemical analysis. MTT assay was carried out to find cytotoxicity. Glucose uptake activity of the extract was carried out using L6 myotubes.

**Results:**

The results showed a strong α-amylase inhibitory activity for the fruit pulp extract of *T.indica* compared to standard acarbose; the IC_50_ of the fruit pulp extract of *T.indica* and acarbose was 34.19 µg/ml 34.83µM. The extract also showed moderate α-glucosidase inhibitory activity. IC_50_ of the fruit pulp extract of *T.indica* and acarbose were 56.91µg/ml and 45.69µM respectively. The cytotoxicity assay showed IC_50_ of >300µg/ml and ≥1000µM for the fruit pulp extract of *T.indica* and metformin. The extract showed 63.99±0.08% glucose uptake in L6 myotubes whereas metformin and insulin at 10µg/ml and 10µM exhibited an uptake of 76.99±0.3% and 84.48±0.45% glucose, respectively.

**Conclusion:**

The study revealed that the fruit pulp extract of *T.indica *Linn does not show any cytotoxic effect and has very good α-amylase and good α-glucosidase inhibitory activities. The glucose uptake potential proves its postprandial hypoglycemic effect. Hence, it may be considered an antidiabetic agent for control of postprandial hyperglycemia.

## Introduction

Diabetes mellitus (DM) is one of the major chronic endocrine disorders, which causes variations in blood glucose levels. Deficit in insulin secretion or action is the main cause of DM. As per the reports of International Diabetes Federation (IDF), more than 415 million people have diabetes worldwide and this number is expected to reach 642 million by 2040 (IDF Diabetes Atlas 2017 ▶). Diabetes is mainly classified into type 1 and type 2 diabetes with type 2 is being the most prevalent. DM is characterized by hyperglycemia and defects in carbohydrate, protein and fat metabolism. Oxidative stress can cause diabetes and diabetes complications. Glucose control plays an important role in maintaining the pro-oxidant/antioxidant balance (Nahar et al., 2014 ▶; Hatanaka et al., 2016 ▶).Currently, insulin secretagogues and sensitizers are used to control hyperglycemia. Nevertheless, carbohydrate digesting enzyme inhibitors are useful in controlling hyperglycemia by reducing glucose absorption from the intestine (Ghosh and Suryawanshi, 2001 ▶; Ghadyale et al., 2012 ▶). Acarbose is commonly used an inhibitor of carbohydrate metabolism in the gastrointestinal tract but it possesses adverse effects such as diarrhea and intestinal disturbances such as bloating, cramping and abdominal pain (Berger, 1985 ▶; Singhet al., 2008 ▶; Mohajeriet al., 2008 ▶). Hence, alternative natural anti-diabetic agents that help to control diabetes are in huge demand.

Recently, the application of natural and herbal medicines has become more prevalent. *T*.*indica *Linn (family *Fabaceae*), is an edible plant. It is widely present in South Asian regions and some portions of Africa. It is commonly cultivated as an ornamental tree and used in making drinks and decoctions used in medicine (Kumar and Bhattacharya, 2008 ▶).Studies revealed the presence of many active constituents such as phenolic compounds, cardiac glycosides, L-(-) malic acid, tartaric acid, the mucilage and pectin, arabinose, xylose, galactose, glucose and uronic acid, in this plant (Bhadoriya et al., 2012 ▶; Kuru, 2014 ▶). The pulp is utilized as a major ingredient in curries, chutneys, sauces, ice-creams and sherbets. The pulp is eaten raw in India and is a source of many micro and macroelements (Ishakuet al., 2016 ▶). This plant elicits hypolipidemic (Lim et al.,2018 ▶), antioxidant (Sandesh et al., 2014 ▶; Reis et al., 2016 ▶), antimicrobial (Escalona-Arranz et al., 2010 ▶), anti-inflammatory and analgesic (Komakech et al., 2019 ▶), antimalarial (Ahmed AOEE and Ayoub SMH,2015 ▶) and hepatoprotectiveactivities (Amir et al., 2016 ▶).Leaves and seeds of *T. indica* were reported to have hypoglycemic activity (Maiti et al., 2005 ▶; Ramachander et al.,2012 ▶). The present study focuses on α-amylase and α-glucosidase inhibitory effect, glucose uptake on L6 myotubes and cytotoxicity profile of the aqueous fruit pulp extract of *T.indica*.

## Materials and Methods

**Plant extract**

The aqueous fruit pulp extract of *T.indica *Linn (product code 4010000759 dated 09/05/2018) was obtained from Synthite Industries Ltd., Kerala, India as a gift. As per the manufacturer's claim, the aqueous extract of the *T.indica* fruit pulp was concentrated under vacuum and the product information states 11-13% of tartaric acid.The product is commercially available.

**Chemicals**

Dinitrosalicyclic acid, 4-nitrophenyl α-D-glucopyranoside, α-glucosidase solution, α- amylase solution (Hi-Media RM638), L6 Monolayer myoblast culture (NCCS, Pune, India, Passage No. 27) penicillin, streptomycin, gentamycin, amphotericin B (Gibco, India). All the chemicals used for this study were of analytical grade.

**Preliminary phytochemical analysis**

To screen the presence of the active principles, the extract was subjected to preliminary phytochemical analysis following standard methods (Harborne, 1973 ▶) (Table 1).

***In-vitro*** **α-amylase inhibitory assay**

*In vitro* amylase inhibition was studied by the method of Bernfeld (Bernfeld P .1955 ▶). Here, 100 μl of the aqueous fruit pulp extract of *T.indica* was allowed to react with 200μl of α-amylase enzyme (Hi media RM 638) and 100 μl of 2 mM of phosphate buffer (pH6.9). After a 20-min incubation, 100μl of 1% starch solution was added. The same was performed for the controls where 200 μl of the enzyme was replaced with the buffer. After incubation for 5 min, 500 μl of dinitrosalicylic acid was added to both control and test. They were kept in a boiling water bath for 5 min. The absorbance was recorded at 540 nm using a spectrophotometer (Shimadzu,Japan) and the percentage inhibition of α-amylase enzyme was calculated using the following formula. A parallel blank contained the reagent mixture without *T.indica* extract.

%inhibition=[(Control-Test)/Control]*100

**α-glucosidase inhibitory activity**

The enzyme inhibition activity for α-glucosidase was evaluated according to the method previously reported by Shibano et al. 1997 with minor modifications (Sanchetia et al., 2011 ▶). The reaction mixture consisted of 50 μl of 0.1 M phosphate buffer (pH 7.0), 25 μl of 0.5 mM 4-nitrophenyl α-D-glucopyranoside (dissolved in 0.1 M phosphate buffer, pH 7.0), 10 μl of the aqueous fruit pulp extract of *T.indica* and 25 μl of α-glucosidase solution (a stock solution of 1 mg/ml in 0.01 M phosphate buffer, pH 7.0 was diluted to 0.1 Unit/ml using the same buffer, pH 7.0 just before assay). This reaction mixture was then incubated at 37°C for 30 min. Then, the reaction was terminated by the addition of 100μl of 0.2 M sodium carbonate solution. The enzymatic hydrolysis of the substrate was monitored by the amount of p-nitro phenol released in the reaction mixture, at 410 nm using a Multimode microplate reader (Perkin Elmer, USA). Individual blanks were prepared for correcting the background absorbance, where the enzymes were replaced with buffer. Controls were assessed in an identical manner by replacing the test extract with methanol. Acarbose was used as a positive control. 

**Cell culture studies**

**Preparation of cell culture**

L6, a monolayer myoblast culture (obtained from NCCS, Pune, India –Passage No- 27) was cultured in DMEM with 10% fetal bovine serum (FBS) supplemented with penicillin (120units/ml), streptomycin (75 µg/ml), gentamycin (160 µg/ml) and amphotericin B (3 µg/ml) in a 5% CO_2_ environment. For differentiation, the L6 cells were transferred to DMEM with 2% FBS for 4 days, post-confluence. The extent of differentiation was established by observing the multinucleate of cells.

**Cytotoxicity assay**

Cytotoxicity of the test extract was assessed by MTT assay (Gohel, 1999 ▶). Cells were plated in 48-well plate at a concentration of 5x10^4^ cells/well. After 24hr of incubation, it was washed with 200 µl of 1x phosphate buffered saline (PBS; pH 7.4) and starved by incubation in serum-free medium for an hour at 37^o^C in CO_2_ incubator. After starvation, cells were treated with different concentrations (1–1000 µg/ml) of the aqueous fruit pulp extract of *T.indica* for 24 hr in serum-free media. At the end of the treatment, media from control and extract-treated cells, were discarded and 50 µl of MTT containing PBS (5 mg/ml) was added to each well. Cells were then incubated for 4hr at 37^o^C in CO_2_ incubator. The purple formazan crystals formed were then dissolved by adding 150 µl of DMSO and mixed effectively by pipetting up and down. Spectrophotometrical absorbance of the purple-blue formazan dye was measured using a Multimode reader (Perkin Elmer, USA) at 570 nm. The optical density of each sample was compared with control optical density and graphs were plotted.

**Glucose uptake assay**

Anti-diabetic activity of the aqueous fruit pulp extract of *T.indica *was assessed in differentiated L6 myotubes using fluorescent tagged 6-NBDG (6-(N-(7-Nitrobenz-2-oxa-1,3-diazol-4-yl) amino)-2-deoxyglucose). L6 myotubes (10,000 cells/well) were seeded in 96-well plates and allowed to confluence around 80%. Then, cells were differentiated using 2% FBS and different concentrations of the extract (1-100 μg/ml) were added. After 24 hr, 5 µl of 10 µM insulin was added to stimulate glucose uptake and incubated for 15 min. Then, 20mg/200ml of 6-NBDG was added and incubated for 10 min in the dark. Glucose uptake in percentage was measured using a Multimode reader (Perkin Elmer, USA) with an excitation/emission filter at 466/540 nm (So Yeon Park et al., 2014 ▶).

**Statistical analysis**

Results are presented as mean±SEM. All tests were performed in triplicate. IC_50_ values were determined by linear regression analysis by Graph Pad software, version 7.

## Results

The preliminary phytochemical screening showed the presence of proteins, saponins, glycosides, alkaloids and anthraquinones (Table 1).

**Table 1:** Preliminary phytochemical analysis

**Chemical constituents**	***T.indica *** ** fruit pulp extract**
Proteins	+
Carbohydrates	-
Reducing sugars	-
Phenolic compounds	-
Tannins	-
Flavonoids	-
Glycosides	+++
Saponins	++
Alkaloid	+++
Steroids	-
Anthraquinones	+++
Quinones	-

[+++highly present; ++moderately present; +mild presence; and -absent]

**Figure 1:**
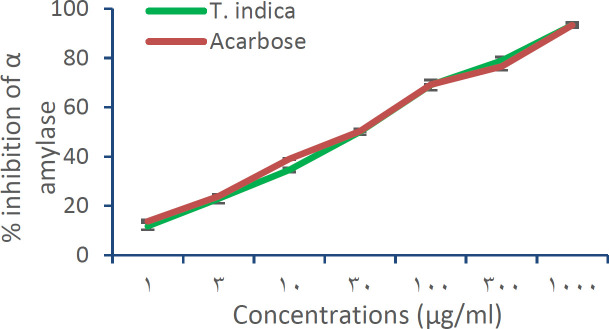
Graphical representation of α-amylase inhibitory activity of the aqueous fruit pulp extract of *T. indica* and acarbose

**Figure 2:**
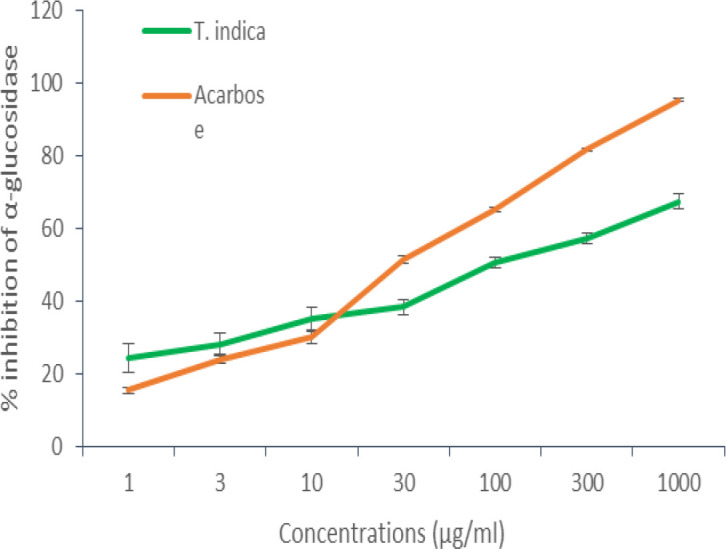
Graphical representation of α-glucosidase inhibitory activity of the aqueous fruit pulp extract of *T. indica* and acarbose

The results showed strong α-amylase inhibitory activity for the test extract compared to standard acarbose (Figure 1). A maximum inhibition of 93±0.60% was achieved at a concentration of 1000 µg/ml by aqueous fruit pulp extract of *T.indica* which was comparable to that of standard acarbose (about 93±1.08%). The IC_50_ of the extract was found to be 34.19 µg/ml and for acarbose 34.83 µM. The maximum α-glucosidase inhibitory activity of the *T.indica* fruit pulp extract was 67±2.17% at 1000µg/ml. Acarbose showed a maximum inhibition of 95± 0.42% at 1000 µM (Figure 2). The IC_50_ of the *T.indica* fruit pulp extract and acarbose were 56.91 µg and 45.69 µM, respectively. 

[All values are expressed as mean±SEM (n=3)]

All values are expressed as mean±SEM (n=3)]

**Figure 3:**
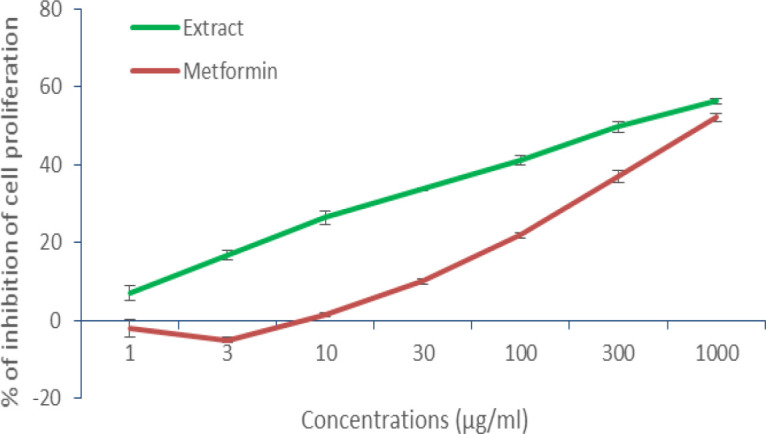
Graphical representation of cytotoxicity (MTT) assay of the aqueous fruit pulp extract of *T. indica* and metformin

The cytotoxicity assay was carried out for the aqueous fruit pulp extract of *T.indica* at different concentrations of 1-1000 µg/ml at a time interval of 24 hr. From the results, it was observed that both extract and standard metformin exhibited a dose-dependent decrease in the % cell proliferation which exhibited ≤50% even at a maximum dose of 1000µg/ml. The IC_50_ of extract and metformin were >300 µg/ml and ≥1000 µM, respectively (Figure 3).

[All values are expressed as mean±SEM (n=3)]

**Figure 4:**
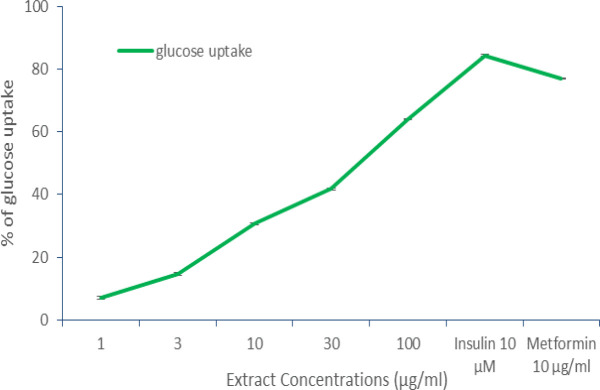
Graphical representation of 6-NBDG glucose uptake potential of the aqueous fruit pulp extract of *T. indica* in L6 myotubes

**Figure 5:**
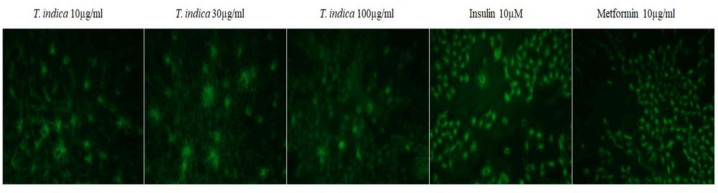
Photosshowing the glucose uptake potential of the aqueous fruit pulp extract of *T. indica* at different concentrations

The glucose uptake potential of the extract was evaluated at different concentrations of 1-100 µg/ml. It was shown found that the extract enhanced glucose uptake in L6 myotubes in a dose-dependent manner which was compared with standard metformin. The maximum percentage of glucose uptake was 63±0.08% at100 µg/ml for the extract while metformin at 10 µg/ml, exhibited 76±0.22% and insulin at 10 µM showed 84.48±0.45% of glucose uptake (Figures 4 and 5).

[All values are expressed as mean±SEM (n=3)]

## Discussion

The phytochemical analysis showed the presence of glycosides, alkaloids and anthraquinones as the major constituents. This result was similar to the findings of Abukakaret al. (Abukakar et al., 2008 ▶). Alkaloids isolated from *Catharanthus roseus *(L.) were reported to increase glucose uptake potential (Tiong et al., 2013 ▶). The anthraquinones, 2-hydroxy-3-methyl-anthraquinone and physcion isolated from *Juncussetchuensis buchen *were confirmed to have hypoglycemic activity, proving its traditional use as an anti-diabetic plant (Cai et al., 2016 ▶). Saponins and glycosides from different plants were also reported to have hypoglycemic and anti-hyperglycemic activity by stimulating insulin release from isolated pancreatic islets (Grover et al., 2002 ▶). Ashwini et al. 2017 ▶ and Gayathri et al. 2018 ▶ also have reported about plants useful to control hyperglycemia and advanced glycatione endproducts.

 In this study, the *T.indica *fruit pulp extract showed a dose-dependent inhibitory effect on both α-amylase and α-glucosidase enzymes with a better effect on α-amylase. Studies showed that inhibition of α-amylase is an effective strategy to reduce postprandial hyperglycaemia for diabetes management (Unnikrishnan et al., 2015 ▶). Different authors reported the antihyperglycaemic effect of different parts of tamarind tree in animal models. Koyaguru et al .reported the antidiabetic activity of the ethanolic extract of the fruit pulp of *T.indica *in alloxan-induced diabetic rats (Koyaguru et al., 2013 ▶). The methanolic extract of fruit pulp and seeds of *T.indica* was reported to have an antihyperglycaemic effect on glucose-induced hyperglycaemic rats (Roy et al., 2010 ▶). The aqueous methanolic extracts of leaf of *T.indica *was also reported to have antidiabetic activity. The possible mechanism behind the antidiabetic activity of the *T.indica* leaf may be the inhibition of free radical generation and subsequent tissue damage induced by alloxan or potentiation of plasma insulin effect by increasing either pancreatic secretion of insulin from existing beta cells or its release (Ramachanderet al., 2012 ▶).

Peripheral tissue especially, skeletal muscle is important to maintain postprandial plasma glucose levels (Klip and Ishiki, 2005 ▶). In postprandial state, 75% of glucose disposal takes place in skeletal muscle (Ehrenborg and Krook, 2009 ▶).Impaired glucose homeostasis occurs due to impaired glucose uptake by skeletal muscles in diabetic Type 2 condition because of insulin resistance (Peppa et al., 2010 ▶). *T. Indica *seed powder was reported to have inhibitory effects on intestinal glucose absorption and significant antihyperglycemic activity in type II diabetic rats (Parvin et al., 2013 ▶). In the present study, the fruit pulp extract showed dose-dependent glucose uptake (Figure 5) .This study throws light on the postprandial hypoglycaemic effect of the aqueous fruit pulp extract of *T.indica*. The α-amylase and α-glucosidase inhibitory effect along with its glucose uptake potential may be the possible mechanism behind the antidiabetic effect of *T.indica* fruit pulp extract.

Here, the plant didnot show any cytotoxicity indicating its safety. Moreover, the fruit pulp is regularly used for culinary purposes. The present study used different *in-vitro *models for antidiabetic activity, therefore, threwlight to on the additional mechanisms involved in the traditional use of this medicinal plant as an antidiabetic agent. Moreover, plant extracts are reported to lack hepatic or renal adverse effects (Mohtashami et al., 2019 ▶). 

The results revealed that the aqueous fruit pulp extract of *T.indica *Linn does not have any cytotoxic effect. The extract not only effectively inhibited the carbohydrate hydrolyzing enzymes such as α-amylase and α-glucosidase, but also enhanced the glucose uptake potential.Hence, the extract may be recommended for the control of postprandial hyperglycemia.
